# Simulation of the Impact of Si Shell Thickness on the Performance of Si-Coated Vertically Aligned Carbon Nanofiber as Li-Ion Battery Anode

**DOI:** 10.3390/nano5042268

**Published:** 2015-12-15

**Authors:** Susobhan Das, Jun Li, Rongqing Hui

**Affiliations:** 1 Department of Electrical Engineering & Computer Science, The University of Kansas, Lawrence, KS 66045, USA; E-Mail: susobhan@ku.edu; 2 Department of Chemistry, Kansas State University, Manhattan, KS 66506-0401, USA; E-Mail: junli@ksu.edu

**Keywords:** Li-ion batteries, battery performance, nanostructures, numerical simulations, silicon anode, carbon nanowires

## Abstract

Micro- and nano-structured electrodes have the potential to improve the performance of Li-ion batteries by increasing the surface area of the electrode and reducing the diffusion distance required by the charged carriers. We report the numerical simulation of Lithium-ion batteries with the anode made of core-shell heterostructures of silicon-coated carbon nanofibers. We show that the energy capacity can be significantly improved by reducing the thickness of the silicon anode to the dimension comparable or less than the Li-ion diffusion length inside silicon. The results of simulation indicate that the contraction of the silicon electrode thickness during the battery discharge process commonly found in experiments also plays a major role in the increase of the energy capacity.

## 1. Introduction

Li-ion battery (LIB) is considered a key component for energy storage in portable electronic devices and systems [1,2]. The energy capacity and power density are important parameters of battery performance [3]. Electrochemical processes of reaction and ion exchange in a LIB not only depend on the material properties of electrode and electrolyte, but also strongly depend on the size and geometric structure of the electrodes [4]. The maturity of micro- and nano-fabrication techniques developed in recent years provided much more flexibilities in battery design, and performance improvements [5,6,7,8]. The reduction of electrode thickness effectively increases the surface area to volume ratio of the electrode which improves ion exchange efficiency between the electrode and electrolyte [9]. In addition, the reduced electrode thickness promotes the penetration of Li-ions thoroughly into the electrode through the diffusion process. Such principles are also applicable to nanostructure geometries such as long nanowires [9] and core-shell materials [10,11]. Understanding the chemical reaction processes and the impact of structural parameters is critically important in battery design and performance optimization. Numerical modeling and simulation allow multi-dimensional optimization of battery performance, which is usually impossible, or much more costly, to be performed experimentally [10]. In this paper we report the results of our numerical simulation of LIBs with the anode made of core-shell heterostructures of coaxially coated silicon on carbon nanofibers.

**Figure 1:**
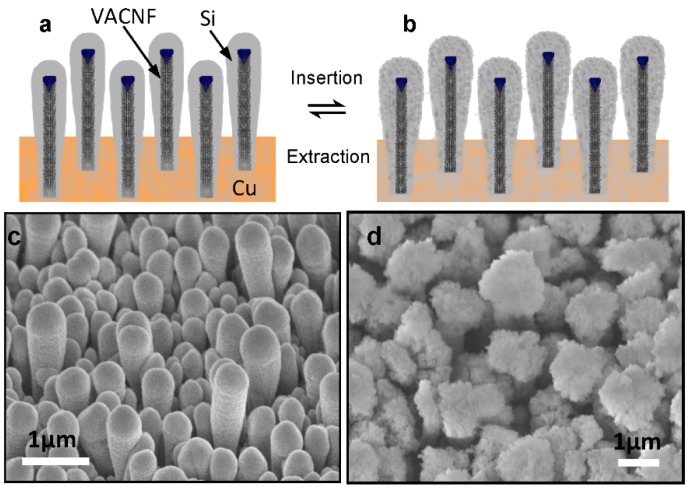
Schematic of Si coating on vertically aligned carbon nanofibers (**a**) before and (**b**) after Lithium insertion, and scanning electron microscopic images showing of the morphology of Si shells in (**c**) in the non-lithiated condition with an average tip diameter of ~460 nm and (**d**) in lithiated conditions with the average tip diameter expanded to ~1.5 µm. All images were taken at 45° perspective view. All scale bars are 1.0 µm. (Reproduced with permission of [16]. Copyright The Royal Society of Chemistry, 2013).

Silicon has been recognized as a very attractive LIB anode material which presents an extremely high theoretical Lithium (Li) storage capacity (4200 mAh/g) and a very low lithiation potential (0.2–0.4 V *vs.* Li/Li^+^) [5,11,12,13]. At room temperature, amorphous Si can provide a maximum capacity of ~3800 mAh/g, ten times that of graphite (372 mAh/g) [13]. However, Li insertion into Si during charging forms alloys, causing up to 320% volume expansion [13,14] which leads to fracturing and loss of electrical connection. Therefore, the capacity of Si thin-film electrode quickly fades after only tens of cycles [15]. Various nanostructured Si materials, particularly Si nanowires, have been demonstrated able to accommodate the mechanical stress and stabilize the electrode besides the advantages of the large specific surface area and short Li^+^ diffusion length in solids [5,13,14]. Hybrid core-shell structures utilizing a highly conductive nanostructured current collector, *i.e.*, vertically aligned carbon nanofibers (VACNFs), coated with silicon shells have shown further enhancement in stability and high-power capability [11]. The contraction (expansion) of silicon shells in the radial direction during Li-ion extraction (insertion) observed in the experiments (see Figure 1) was attributed as the main reason for the improved performance [16].

Various simulation and modeling work have been carried out in order to gain mechanistic insights of the experimental observations. Soni *et al.* have investigated the lateral volume expansion and stress generation during lithiation of micro-islands (7 µm, 17 µm, and 40 µm squares) of thin amorphous Si film on a flat Ti current collector [17]. A continuum model describing the stress accommodation due to interfacial sliding has been used to explain the experimentally measured stresses. The results indicated that engineering the proper Si island size is effective in mitigating the lithiation-induced stress and mechanical degradation in Si LIB anodes. However, the core-shell structure down to the relevant Li diffusion length in Si (*i.e.*, ~1 µm) at normal charge-discharge rates has not been investigated. Here we developed an approach to study the system of core-shell nanowires using a COMSOL [18] numerical simulation module. Our simulation indicates that the contraction (expansion) of silicon shell during the discharge (charge) process plays an important role contributing to the LIB energy capacity increase.

## 2. Simulation Model

**Figure 2:**
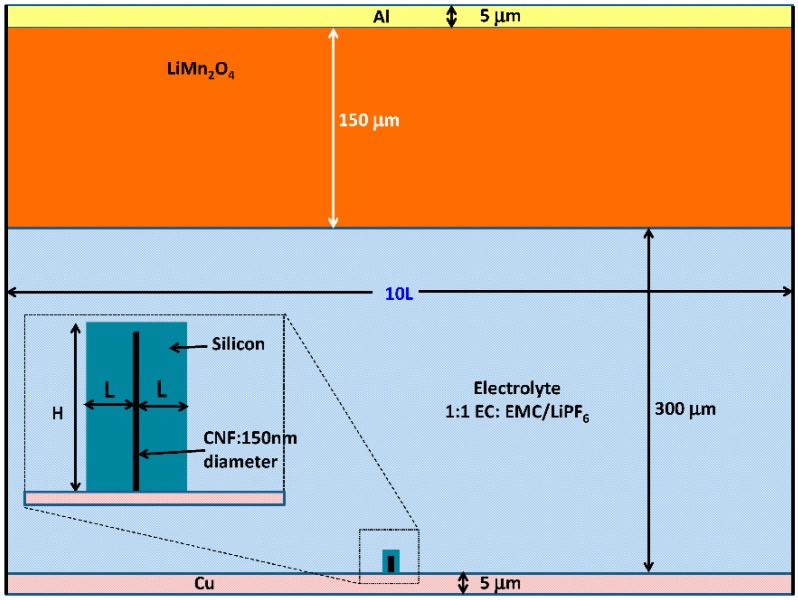
Simulation Schematic (inset shows details of the silicon anode coated on carbon nanofiber (CNF)).

Considering the LIB structure based on silicon-coated carbon nanofibers as the anode [11,16], the schematic of 2D simulation structure used in this study is described in Figure 2 based on the COMSOL Li-ion battery simulator. The length of the vertically aligned carbon nanofiber (CNF) attached on a Cu foil is *H* = 30 µm with a diameter of 150 nm, which is surrounded by silicon. Throughout the simulation, the thickness *L* of the silicon coated on each side of CNF is varied from 1 µm to 30 µm at the beginning of discharge, and the thickness of silicon on top of the CNF is fixed at 0.2 µm.

As the major focus of this work is on the micro-/nano- structured silicon anode, the volume of cathode material (LiMn_2_O_4_) is chosen to be large enough so that the Li storage capacity is not limited by the cathode. As shown in Figure 2, the height and width of the cathode materials are 150 µm and 10*L*, respectively, with a separation of 300 µm from the bottom of the anode. A mixture of ethylene carbonate (EC) and ethyl methyl carbonate (EMC) at a volume ratio of 1:1 dissolved with 1.0 M LiPF_6_ is used as the electrolyte which fills the entire space between the anode and the cathode. The CNF core of the anode is directly connected to 5 µm thick copper (Cu) foil as the current collector, and the current collector on the cathode side is aluminum (Al) of also 5 µm in the thickness. To simplify the simulation, only one silicon anode coated on CNF is considered, and the width of the simulation area is 10*L*. This is equivalent to a periodical structure with a separation of 10*L* between adjacent silicon wires.

**Figure 3:**
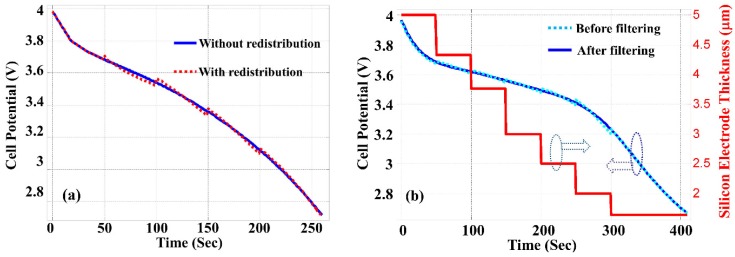
(**a**) Discharge graph with *L* = 5 µm, *H* = 30 µm, and a constant current density of 4000 A/m^2^ without considering thickness contraction of silicon layer. Dotted line: calculated with time-sectioning and redistribution of electrolyte salt concentration at the beginning of each time window. Solid line: calculated with standard COMSOL [18] procedure without time-sectioning. (**b**) Same condition as (**a**) but with thickness contraction of silicon layer indicated by the right axis. Dotted line: calculated with time-sectioning, redistribution of electrolyte salt concentration and thickness reduction at the beginning of each time window. Solid line: a smoothed best fit to the dotted line.

It has been experimentally observed that the volume of the silicon anode could expand by as much as 320% during the charging process, and shrink by the same amount during discharge [14]. It is thus necessary to include this expansion/contraction in the simulation. The current COMSOL simulation software does not straightforwardly allow the continuous variation of the geometric dimension of the electrode during the simulation of charge and discharge processes. Therefore we have to divide the continuous process into discrete sections so that the thickness of the silicon anode can be reset at the beginning of each time window. This requires both the level and the distribution of electrolyte salt (*i.e.*, Li^+^) concentration inside the silicon layer at the beginning of each time window to be the same as those at the end of the previous window. As the simulation software only allows a constant level of electrolyte salt concentration across the silicon layer, we use the spatially averaged concentration of electrolyte salt at the end of the previous time window as the initial level for the simulation of the subsequent time window. As a consequence, in the discharge process a slight discontinuity is created in the calculated cell potential between adjacent time windows. For the purpose of evaluating the accuracy of this approach, we calculated the cell potential as the function of time in the discharge process with a constant current density of 4000 A/m^2^ and assume a constant thickness of the silicon layer *L* = 5 µm. The length of the silicon anode wire is *H* = 30 µm. The dotted line in Figure 3a shows the results obtained when the discharge process is divided into 6 time windows, and the electrolyte salt concentration is redistributed at the beginning of each time window as discussed above. The solid line in Figure 3a is obtained with the standard simulation procedure without timing windowing, which is possible because the effect of silicon thickness contraction is not considered in this calculation. The reasonably good agreement between these two curves in Figure 3a justifies the use of this approximation. Except for the slight (<2%) discontinuity at the beginning of each time window, the result obtained with time windowing and redistribution of electrolyte concentration does not introduce unacceptable errors.

While Figure 3a is shown only for the purpose of modeling validation, Figure 3b shows the cell potential as the function of discharging time calculated with the contraction of the silicon anode thickness. To consider the anode thickness contraction in the discharge process, at the end of each time window the thickness of the silicon anode is reduced by the amount proportional to the peak value of electrolyte salt concentration increase without contraction, and the average value of the electrolyte salt concentration is used as the initial condition for the simulation of the next time window. The discontinuity between sections shown by the dotted line in Figure 3b is the consequence of this windowing process. The solid line in Figure 3b is obtained by best fitting to the calculated data through a smoothing filter. The comparison between Figure 3a,b indicates that the contraction of silicon anode during the LIB discharge process has the effect to increase the energy capacity shown as the prolonged discharging time (410 s *vs.* 260 s).

**Figure 4:**
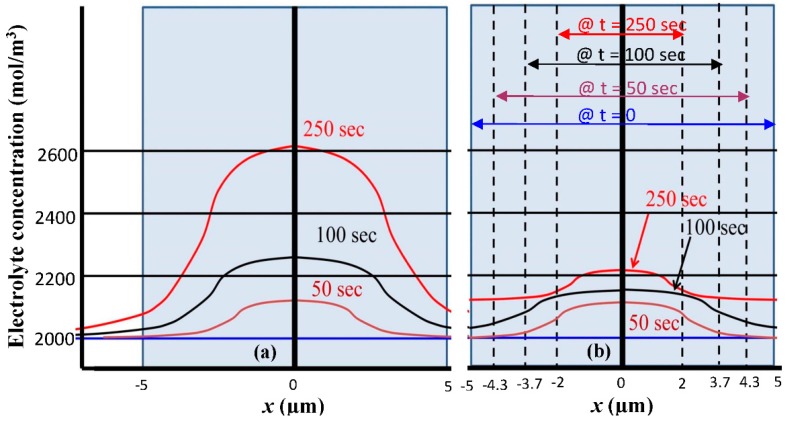
Distribution of electrolyte salt concentration within silicon anode at different times of discharge process (**a**) without and (**b**) with thickness contraction.

To further explain the procedure used in the simulation to consider the contraction of silicon anode thickness, Figure 4a shows the electrolyte salt distribution inside the silicon anode with a thickness of *L* = 5 µm. The electrolyte salt concentration is 2000 mol/m^3^ (*i.e.*, 2 M) which is uniform in both the solution and the Si anode when the LIB is fully charged. This electrolyte salt concentration level increases along the process of discharge and the distribution across the silicon anode becomes non-uniform as shown in Figure 4a. To consider the size contraction effect in the discharge process, we assume that the thickness *L* of the silicon anode is inversely proportional to the concentration of electrolyte salt, and *L* is decreased by approximately 3 times when the peak electrolyte salt concentration increases from 2000 mol/m^3^ to 2610 mol/m^3^. The silicon anode thickness contraction as the function of discharge time is shown in Figure 3b, which is a discrete function assuming the thickness contraction only happens at the end of each time section. Figure 4b shows the actual electrolyte salt concentration when silicon anode thickness contraction is considered. Dashed vertical lines in Figure 4b indicate the boundaries of the contracted silicon electrode corresponding to each time window. To obtain Figure 4b, at the end of each time section, the average electrolyte salt concentration is calculated and used as the initial condition of the next time section with a reduced thickness of silicon anode. The reduction of silicon anode thickness helps reducing the accumulation of electrolyte salt, and as the result both the electrolyte salt concentration level increase and the variation of electrolyte salt across the anode are reduced in the discharge process in comparison to the case without the thickness contraction. This helps slowing down Li ion building up in the silicon anode, which improves the LIB energy capacity.

## 3. Simulation Results and Discussion

**Figure 5:**
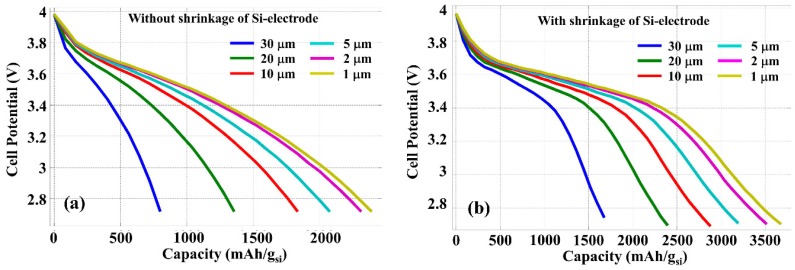
Discharge graph calculated with different thickness *L* of silicon anode and with a constant discharge current density of 4000 A/m^2^, without (**a**) and with (**b**) the contraction of silicon anode.

Based on the simulation model presented in the last section, the performance of LIB with different thicknesses of silicon anodes is investigated with and without thickness contraction in the discharge process. Figure 5 shows the cell potential variation as the function of the specific Li^+^ extraction capacity (relative to the mass of Si) calculated with a constant current density of 4000 A/m^2^. *H* = 30 µm was assumed for the length of the anode. Results shown in Figure 5a,b are obtained, respectively, without and with considering the contraction of silicon anode thickness during discharge. With a constant current density, the reduction of silicon anode thickness is equivalent to a reduced current contribution from each silicon wire, because an increased number of silicon wires can be inserted into the same cross section area. Figure 5 indicates that high specific Li^+^ extraction capacity can be obtained with smaller silicon anode thickness because of the more efficient chemical reaction and Li^+^ ion penetration from electrolyte into the silicon. This Li^+^ specific extraction capacity improvement due to the reduced silicon thickness saturates when this thickness approaches or smaller than the diffusion length of Li^+^ ions in silicon, which is on the order of a few micrometers. In addition, the contraction of silicon anode thickness in the discharge process further facilitates the Li^+^ ion diffuse out from the electrode resulting in more efficient chemical reaction, and thus the specific energy capacity is significantly increased as can be easily seen through the comparison between Figure 5a,b.

Note that in the simulation using COMSOL software, the initial Li-ion density is chosen in such a way that the cell potential at fully charged condition reaches to 4 V and the maximum specific capacity of silicon can be achieved as 3800 mAh/g_Si_ at room temperature, which provides the full Li^+^ storage capacity available by the LIB. Figure 5b shows that the contraction of silicon electrode thickness during the discharge process helps battery to reach the full Li^+^ extraction capacity of Si.

**Figure 6:**
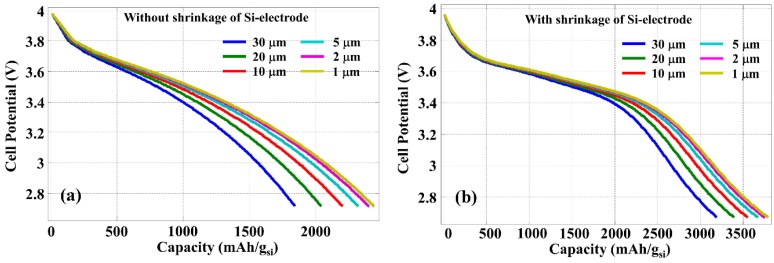
Discharge graph calculated with different thickness of silicon anode and with a constant discharge current density of 400 A/m^2^, (**a**) without and (**b**) with the contraction of silicon anode.

As the capacity improvement with reduced silicon thickness is due to the more efficient diffusion of Li-ion into silicon, the discharge rate determined by the current density should also play an important role. Figure 6 shows the cell potential as the function of power capacity calculated with a constant discharge current density of 400 A/m^2^, which is 10 times lower than that used for Figure 5. Because the discharge process is slower with lower current density, Li-ion diffusion is more efficient even with a thicker silicon layer, thus the improvement due to the reduced silicon thickness is less significant. However, the contraction of silicon layer thickness during the discharging process is still effective in the increase of LIB energy capacity.

**Figure 7:**
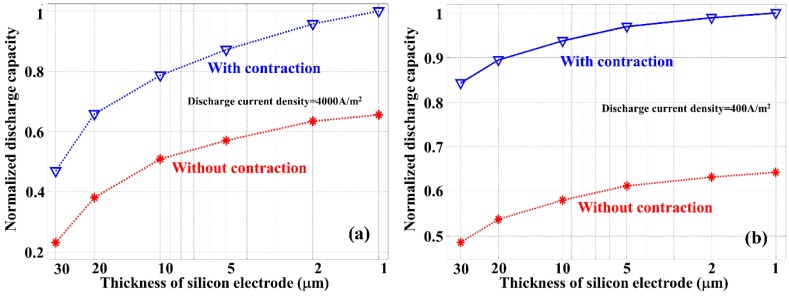
Normalized discharge capacity calculated with different thickness of silicon anode with (open triangles) and without (stars) considering the contraction of silicon anode. Discharge current densities used in the simulations are (**a**) 4000 A/m^2^ and (**b**) 400 A/m^2^.

To better understand the impact of silicon anode thickness and the benefit introduced by thickness contraction in the discharge process, Figure 7 shows the normalized discharge capacity as the function of silicon anode thickness for the discharge current densities of 4000 A/m^2^ (Figure 7a) and 400 A/m^2^ (Figure 7b), respectively. Under a constant discharge current density, the discharge capacity was obtained by integrating the cell voltage as the function of time throughout the discharge process. Figure 7 indicates that the normalized discharge capacity increases monotonically in all cases with the decrease of the silicon anode thickness. However, the saturation of this capacity improvement depends on the discharge current density. Higher current density requires a faster Li-ion diffusion rate so that a reduced silicon anode thickness is more beneficial. On the other hand, a relatively low current density requires a lower carrier diffusion rate, and thus the benefit introduced through silicon thickness reduction is less significant. The most important observation of Figure 7 is that for both current densities, the contraction of silicon anode in the discharge process is responsible for more than 35% improvement in the energy capacity, which has to be considered in LIB design and performance optimization.

In the LIB discharge process, the electrolyte salt concentration is a key parameter, and an increased level of electrolyte salt concentration inside the silicon electrode reduces the electrical potential of the battery. A smaller thickness of silicon anode helps limiting the increase of electrolyte salt concentration in the discharge process, especially when the discharge current density is large. The contraction of the silicon anode thickness during the discharge process further reduces the increase of electrolyte salt concentration which is responsible for an increased discharge capacity of the battery.

Note that in the simulation we have assumed an ideal case of uniform coating of silicon on CNFs, so that the diffusion of Li-ions into silicon anode is primarily a one-dimensional problem in the horizontal direction. In this case, increasing the length of CNF would linearly increase the areal current density due to the increase of the silicon volume. Although experimentally realizing uniform coating of silicon on long CNF is still challenging so far, its potential benefit in increasing the total energy storage capacity of LIB is evident.

**Figure 8:**
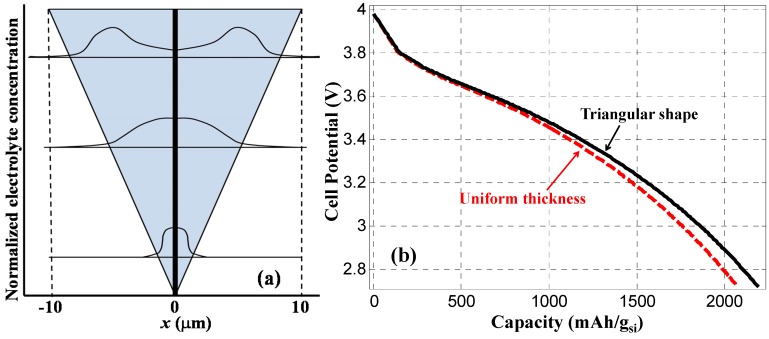
(**a**) Triangle shaped silicon anode (shaded area) coated on 30 µm long CNF, and normalized distribution of electrolyte salt concentration calculated at within silicon anode at different times of discharge process (**a**) without and (**b**) with thickness contraction 250 s after the start of discharging at three different cross sections along the CNF (3 µm, 15 µm, and 24 µm, respectively, from the bottom). (**b**) Discharge graph calculated with a constant discharge current density of 4000 A/m^2^ for triangle anode shape (solid line) and uniform anode thickness with the same silicon volume.

To illustrate the impact of non-uniform coating of silicon on the CNF, we have performed a simulation based on the structure shown in Figure 8a, where the thickness of silicon coating linearly increased from 0 to 10 µm along a 30 µm long CNF. Normalized electrolyte salt concentrations were calculated 250 s after the start of discharging at three different cross sections along the CNF (3 µm, 15 µm, and 24 µm, respectively, from the bottom). The electrolyte salt concentration is assumed uniform in both the solution and the Si anode when the LIB is fully charged. This concentration level increases along the process of discharge, and the distribution of concentration across silicon anode also depends on the thickness of the silicon layer. Li^+^ ion penetration from electrolyte into the silicon is easier with thinner silicon layer, which results in more efficient chemical reaction near the bottom of the CNF. Whereas, near the top of the CNF with thicker silicon coating, the volume efficiency of chemical reaction become relatively low. The solid line in Figure 8b shows the calculated cell potential as the function of discharge capacity for the triangular silicon anode shown in Figure 8a with a constant discharge current density of 4000 A/m^2^. As a comparison, the dashed line in Figure 8b was calculated based on a silicon anode of the same length but with a uniform thickness of *L* = 5 µm. Although silicon anode thickness contraction during the discharge process was not considered, Figure 8b indicates that for the same silicon volume, the non-uniform coating of silicon layer reported in our previous experiment [16] would not necessarily degrade the discharge capacity.

## 4. Conclusions

We have numerically simulated the performance of Li-ion batteries with the anode made of core-shell heterostructures of silicon-coated carbon nanofibers. The results of our simulation indicate that the energy capacity of LIB can be significantly improved by reducing the thickness of the silicon wires to the dimension comparable or less than the Li-ion diffusion length inside silicon. We also demonstrated that the contraction of the silicon thickness during the discharge process played a major rule contributing to the increase of LIB energy capacity. Both the reduced silicon anode thickness and the contraction of the silicon layer in the discharge process help reducing the electrolyte salt accumulation inside the silicon and result in more efficient Li^+^ ion diffusing out from silicon.
